# Household Hints and Recipes

**Published:** 1882-04

**Authors:** 


### Household Hints & Recipes

WATERPROOF CANVAS FOR COVERING CARTS, ETC.—9½ gallons linseed oil, 1 lb. litharge, 1 lb. umber, boiled together for 24 hours. May be colored with any paint. Lay on with a brush.

---

TO COLOR IRON BLACK.—A brilliant black is produced on iron and steel by applying, with a fine hair brush, a mixture of turpentine and sulphur boiled together. When the turpentine evaporates, there remains on the metal a thin layer of sulphur, which unites closely with the iron when heated for a time over a spirit or gas flame. This varnish protects the metal perfectly, and is quite durable.

---

NEW METHOD OF EMBALMING BODIES AND PRESERVING TISSUES.—Dr. Virodtxeff (*Balsamirovanie*, pp. xi, 164, St. Petersburg, 1881) recommends the following preparation as an efficient agent in the embalming of bodies and the preservation of tissues:

Thymol, 5 parts.
Alcohol, 45 parts.
Glycerine, 2,160 parts.
Water, 1,081 parts.

It is cheap, inocuous, free from unpleasant odor, possesses the property of keeping the body soft, elastic, fresh and life-like, and does not ruin instruments. Thymol is selected as being superior to other antiseptics, and glycerine is added, both on account of its own preservative qualities and to retard the evaporation of the fluid. For the preparation of tissues the same solution is employed. If the cadaver be quite lean, or the tissues very delicate, equal parts of water and glycerine (1,620 of each) are combined with the above quantities of thymol and alcohol. To inject a body, half its weight of the fluid is necessary. A properly embalmed cadaver may be preserved indefinitely under ordinary circumstances, gradually shrinking and mummifying without putrefaction. Specimens are either to be injected or macerated in this fluid. Maceration must not be too prolonged—the appearance of the specimen should act as a guide. The part, after having been thoroughly cleansed with water and prepared, and may then be exposed for months to the air without losing its consistency, form and color. Permanent specimens may be enclosed in a hermetically sealed glass vessel containing a little of the same solution. Dr. Peabody has used this preserving fluid with excellent results in the New York Hospital Museum. —*Medical Record*.

---

DYEING BILLIARD BALLS.—

1. *Black*. Boil for a short time in a strained solution of logwood, afterwards immerse them in a solution of green sulphate of iron.

2. *Blue*. Immerse for a short time in a dilute solution of indigo-carmine.

3. *Yellow, a*. Immerse for a few minutes in water containing a little stannous chloride (protochloride of tin); afterwards in a hot, strained decoction of fustic.

*b*. Immerse for about fifteen minutes in a solution of chromate of potassium.

4. *Orange*. Treat as in the first process for yellow, but add to the fustic some shavings of Brazil-wood (Pernambaco-wood).

5. *Red, a*. Macerate cochineal in vinegar, and boil the balls in the liquid for a few minutes.

*b*. Carmine dissolved in ammonia may be used. The tint is more purple-red.

*c*. Immerse in a very dilute solution of stannous chloride, and afterwards in a boiling solution of Brazil-wood. A little fustic turns the color to scarlet.

*d*. Ivory dyed as last directed, is rendered cherry-red by immersion in a very dilute solution of potash.

*e*. Immerse in an alcoholic solution of alizarin-paste.

6. *Violet, a*. Dye red first; then immerse for an instant in solution of indigo-carmine.

*b*. Macerate in dilute solution of stannous chloride; then dip into a hot decoction of logwood. If the balls be afterwards laid in water acidulated with a few drops of nitric acid, the color, turns purple red.

7. *Green, a* Dye first yellow, and afterwards dip into solution of indigo-carmine.

*b*. Place the balls for several hours in concentrated solution of chromate of potassium; afterwards expose to sunlight; this produces a dark, bluish-green tint.

N. B.—Unpolished ivory takes color better than polished. Ivory must not be boiled long in liquids, and when taken out of hot liquid, should be rapidly cooled by laying in cold water (according to Karmarsch). Ivory is very sensitive to change of temperature, and while it will stand the treatment just mentioned, will not stand forcible blows when cold.

